# Depleting Components of the THO Complex Causes Increased Telomere Length by Reducing the Expression of the Telomere-Associated Protein Rif1p

**DOI:** 10.1371/journal.pone.0033498

**Published:** 2012-03-20

**Authors:** Tai-Yuan Yu, Chen-Yi Wang, Jing-Jer Lin

**Affiliations:** 1 Institute of Biopharmaceutical Sciences, National Yang-Ming University, Taipei, Taiwan, People's Republic of China; 2 Institute of Biochemistry and Molecular Biology, National Taiwan University College of Medicine, Taipei, Taiwan, People's Republic of China; Tulane University Health Sciences Center, United States of America

## Abstract

Telomere length is regulated mostly by proteins directly associated with telomeres. However, genome-wide analysis of *Saccharomyces cerevisiae* mutants has revealed that deletion of Hpr1p, a component of the THO complex, also affects telomere length. The THO complex comprises four protein subunits, namely, Tho2p, Hpr1p, Mft1p, and Thp2p. These subunits interplay between transcription elongation and co-transcriptional assembly of export-competent mRNPs. Here we found that the deletion of *tho2* or *hpr1* caused telomere lengthening by ∼50–100 bps, whereas that of *mft1* or *thp2* did not affect telomere length. Since the THO complex functions in transcription elongation, we analyzed the expression of telomere-associated proteins in mutants depleted of complex components. We found that both the mRNA and protein levels of *RIF1* were decreased in *tho2* and *hpr1* cells. *RIF1* encodes a 1917-amino acid polypeptide that is involved in regulating telomere length and the formation of telomeric heterochromatin. Hpr1p and Tho2p appeared to affect telomeres through Rif1p, as increased Rif1p levels suppressed the telomere lengthening in *tho2* and *hpr1* cells. Moreover, yeast cells carrying *rif1 tho2* or *rif1 hpr1* double mutations showed telomere lengths and telomere silencing effects similar to those observed in the *rif1* mutant. Thus, we conclude that mutations of components of the THO complex affect telomere functions by reducing the expression of a telomere-associated protein, Rif1p.

## Introduction

Telomeres are the structure at the ends of eukaryotic linear chromosomes [1], [2]. They are essential for the maintenance of chromosome integrity, and protect natural DNA ends from being recognized as double-strand breaks. In most organisms, the telomeric DNA is composed of short, tandemly repeated sequences with a strand rich in guanine residues (G-strand) running 5′ to 3′ toward the end of the telomere. For example, the telomeric sequences in the brewer's yeast *Saccharomyces cerevisiae* are ∼250–300 base pair-long TG_1–3_/C_1–3_A repeats. Telomeres are maintained by telomerase in most eukaryotes [3]. Telomerase is a ribonucleoprotein containing a catalytic protein component *TERT* (telomerase reverse transcriptase) and an associated RNA moiety, *TER*, which serves as the template to extend telomeric DNA sequences. In *S. cerevisiae*, these two components are encoded by *EST2* [4], [5] and *TLC1* [6], respectively.

Telomere length homeostasis involves the coordination of telomere lengthening and shortening processes. Although the complete mechanism of telomere length regulation remains to be elucidated, controlling telomerase activity appears to be a major part of the process [7]. For example, the binding of telomerase Tlc1 RNA by yeast Ku proteins contributes to telomere length regulation [8]. The mutation of *YKU70* or *YKU80* causes shortening of telomere length [9]–[14]. It has also been reported that Pif1p helicase negatively regulates telomerase by removing telomerase from the telomeric DNA [15], [16]. As a consequence, mutation of *PIF1* has been shown to lengthen telomeres [17]. These telomere-associated proteins affect telomere length by directly regulating the telomerase activity [7]. Regulation of telomerase could also be achieved through posttranslational modification of telomere-associated proteins. For example, phosphorylation of the telomere binding protein Cdc13p by DNA damage response kinases Tel1p/Mec1p is required for recruiting telomerase onto telomeres for telomere replication [18]. Short telomeres have been observed in cells missing either *TEL1* or *MEC1* [19]–[21].

Telomere length homeostasis can also be regulated by the repressor/activator protein 1, Rap1p. in *S. cerevisiae* [22]. It has been reported that several *rap1* temperature-sensitive mutants display phenotypes consistent with the role of Rap1p as a negative regulator of telomere length [23]–[25]. Rap1p binds to a loosely defined recognition site within the double-stranded TG_1–3_ telomeric DNA tracts to affect telomere functions [23]–[25]. Regulation of telomere length by Rap1p appears to be mediated by Rif1p and Rif2p, which bind to the protein-interaction domain at the C-terminus of Rap1p. Deletion of the nonessential *RIF1* and *RIF2* genes results in extensive telomere elongation [26], [27]. Studies of *Kluyveromyces lactis* have further confirmed the role of Rap1p in regulating telomere length [28]–[30].

In addition to affecting telomerase and telomere-associated proteins, genes affecting telomere length have also been identified as being involved in diverse cellular functions [31]–[33]. Among them, deletion of *HPR1* has been shown to increase telomere length by ∼50–150 bp. *HPR1* is a component of the THO complex (a suppressor of the *T*ranscriptional defects of *H*pr1 mutants by *O*verexpression) that is involved in transcriptionelongation and the export of transcribed mRNAs [34], [35]. The THO complex is a conserved nuclear complex that is formed by four protein subunits: Hpr1p, Tho2p (Rlr1p), Mft1p, and Thp2p in *S. cerevisiae*. Mutations in THO components cause defects in transcription-dependent hyper-recombination and mRNA export [36]–[39]. Moreover, there is evidence that DNA∶RNA hybrids are formed co-transcriptionally, resulting in impaired transcription elongation of GC-rich and long DNA sequences and defective transcription-associated recombination [40]. However, it is unclear how mutations of THO components cause changes in telomere length. Here we found that mutations of THO components affect telomeres by reducing the expression of Rif1p. Our results reveal a novel mechanism by which telomere functions could be affected indirectly through perturbing the expression of a telomere-associated component.

## Results

### Mutations of THO complex components *HPR1* and *THO2* affect telomere length

**Figure 1:**
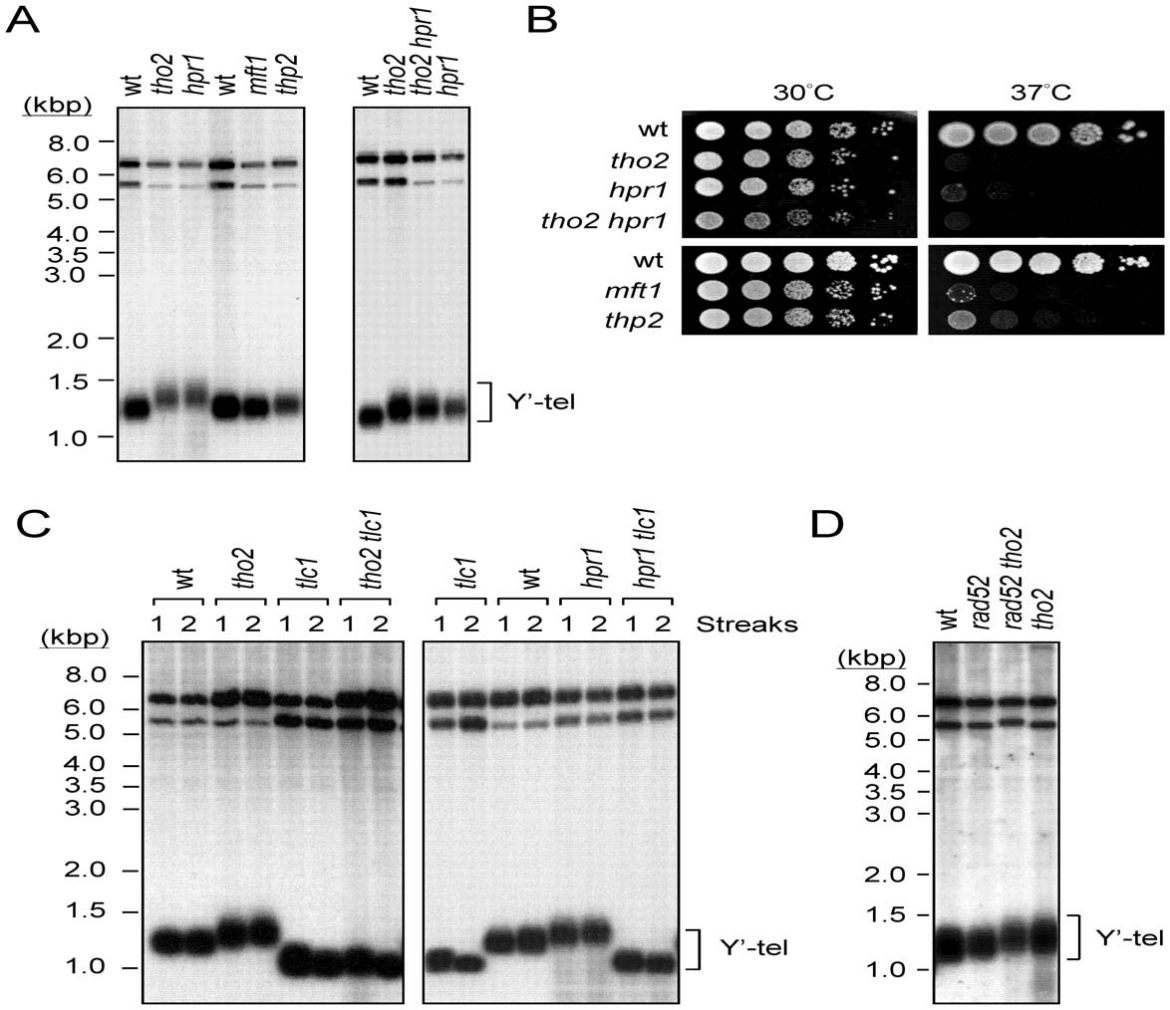
THO complex components Hpr1p and Tho2p affect telomere length. (**A**) Telomere lengthening in *hpr1* and *tho2* strains. Yeast DNA was isolated from the YPH499 yeast strain with indicated mutations, digested with *Xho*I, separated on a 1% agarose gel and analyzed by Southern blotting. The blots were hybridized with probes prepared using the 600-bp sequence from the Y′ element 3′ end. The position of the Y′ telomere is indicated. (**B**) Temperature-sensitive growth of THO complex mutants. Aliquots of ten-fold serial dilutions of yeast cells were spotted on YEPD plates and incubated at 30 or 37°C for three days. (**C**) Telomerase is required for the telomere lengthening effects in *hpr1* and *tho2* cells. Yeast cells carrying the indicated mutations were freshly sporulated and grown on YEPD plates at 30°C after 1 or 2 restreaks. Total yeast DNA was then isolated and analyzed by Southern blotting. (**D**) Recombination is not required for telomere lengthening in *tho2* cells. Total yeast DNA from the indicated strains was isolated and analyzed by Southern blotting.

In a genome-wide screen, mutation of *HPR1* was found to cause a ∼50–150 bp increase in telomere length [31]. Since Hpr1p is a component of the THO complex, we first tested whether the other three THO components also affect telomere length. In *S. cerevisiae*, the middle repetitive sequences known as the Y′ elements are found in the subtelomeric regions of most chromosomes [41]. In wild-type cells, *Xho*I digestion produces a ∼1.3 kbp fragment from the ends of Y′-bearing chromosomes that contains ∼870 bp of Y′ and the terminal tract of ∼350–430 bp of TG_1–3_/C_1–3_A DNA. However, the lengths of Y′-bearing telomeres in both *hpr1* and *tho2* cells appeared ∼75 bp longer than those in wild-type cells (Fig. 1A). This telomere length phenotype was not progressive, as increased cell divisions did not further affect its length (data not shown). Interestingly, mutations of the other two THO complex components, *MFT1* and *THP2*, did not affect telomere length (Fig. 1A), although it has been reported that mutations of these two genes cause slightly shorter telomeres [31]. The reason for the apparent discrepancy is unclear. Telomere lengths of yeast cells harboring both *hpr1* and *tho2* mutations were also analyzed. As shown in Figure 1A (right panel), *hpr1 tho2* double mutations did not further increase the telomere length. Thus, *HPR1* and *THO2* might affect telomere length through the same pathway. As a control, the temperature sensitivity of the resulting strains were analyzed since mutations of THO components also exhibit severe growth defects at 37°C [42], [43]. Indeed, all of the THO mutant cells showed the expected temperature-sensitive phenotype (Fig. 1B).

To determine if the telomere lengthening in *hpr1* and *tho2* cells required the telomerase activity, deletion of telomerase RNA component *TLC1* was introduced into *hpr1* and *tho2* cells, respectively. Yeast cells losing telomerase RNA undergo progressive telomere shortening which eventually leads to cell death [6]. As shown in Figure 1C, telomere lengths in *hpr1 tlc1* and *tho2 tlc1* cells were similar to those in *tlc1* cells. The results indicate that the long telomeres observed in *hpr1* and *tho2* cells required functional telomerase. The effect of recombination on telomere lengthening of *hpr1* and *tho2* cells was also analyzed. A *rad52* mutation was introduced into *tho2* cells and telomere length was examined. As shown in Figure 1D, *rad52* did not affect telomere length in either wild-type or *tho2* cells. The results suggest that telomere lengthening observed in THO complex mutants is not due to telomere recombination.

### *RIF1* expression is reduced in *hpr1* and *tho2* cells

**Figure 2:**
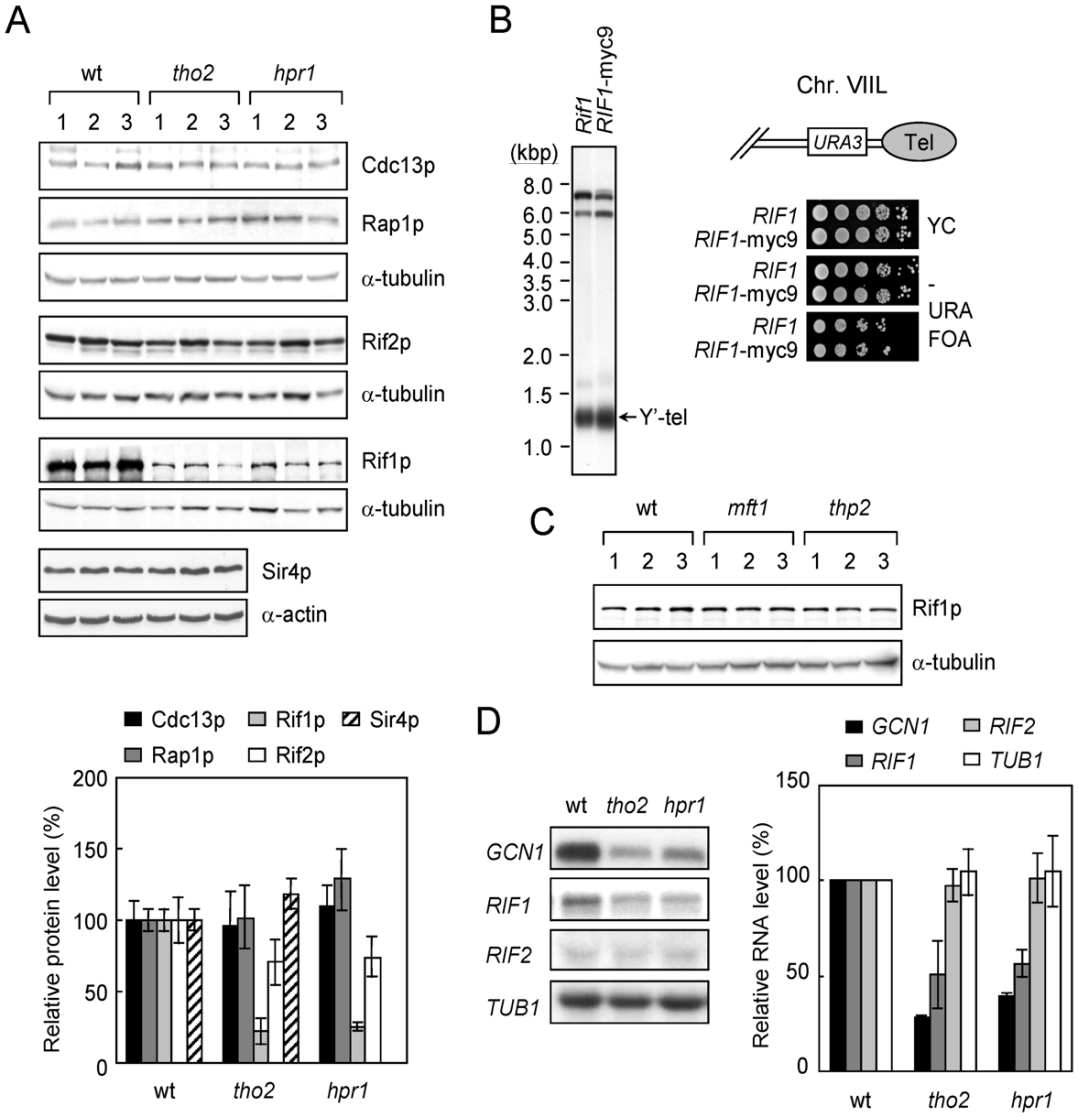
*tho2* and *hpr1* selectively decrease the expression level of Rif1p. (**A**) The Rif1p protein level is decreased in *tho2* and *hpr1* cells. Total yeast proteins from wild-type, *tho2* or *hpr1* yeast cells were precipitated with TCA, separated on an 8% SDS-polyacrylamide gel, and analyzed by immunoblotting using antibodies against Rap1p, Cdc13p, α-tubulin, α-actin, Myc (for myc9-tagged Rif1p and Rif2p), or TAP (for TAP-tagged Sir4p) (top panel). The percentages of protein expression relative to the wild-type cells are quantified (bottom panel). The bars were standard deviations determined using data from three different colonies of the indicated yeast strain. (**B**) The myc9-epitope tagging does not affect the effect of Rif1p on telomere length and telomere silencing. Telomere length of wild-type *RIF1* or *RIF1-myc9* cells were determined by Southern blotting using the Y′ element probe (left panel). Telomere silencing effects were determined in *RIF1* or *RIF1-myc9* cells. Yeast cells in 10-fold serial dilutions were spotted on YC, YC lacking uracil, or plates containing 5-FOA (right panel). (**C**) The Rif1p protein level is not affected in *mft1* and *thp2* cells. The Rif1p level is analyzed in *mft1* and *thp2* cells using immunoblotting analysis. (**D**) *tho2* and *hpr1* reduced the *RIF1* RNA levels. The RNA transcripts of *GCN1*, *RIF1*, *RIF2*, and *TUB1* were analyzed using Northern blotting assays (left panel). The expression levels of the indicated RNA transcripts were quantified and displayed as the percentages relative to the expression level in wild-type cells (right panel). The error bars were standard deviations calculated using data from three different colonies of the indicated yeast strain.

Next, we explored the mechanism by which *HPR1* and *THO2* affect telomere length. The THO complex is involved in transcription elongation. Hybrids of template DNA and transcribed RNA are formed during transcription in mutants of the THO complex components [40]. As a result, the transcription of long genes is preferentially repressed [39], [44]. We hypothesized that mutations of *HPR1* and *THO2* might affect telomere length by influencing the expression of genes that encode telomere-associated proteins. We first surveyed the length of telomere-associated proteins (Table 1). Among those we examined, *RIF1* is the longest gene with a size of 5,751 bps that encodes a protein with 1,916 amino acid residues. To test if *hpr1* and *tho2* affect the expression of the long genes, we analyzed the Rif1p protein level in mutant cells. As shown in Figure 2A, Rif1p levels were decreased by ∼4 folds in both *hpr1* and *tho2* cells. In comparison, *SIR4* (4,077 bps), *CDC13* (2,775 bps), *RAP1* (2,484 bps), and *RIF2* (1,188 bps) were also analyzed. The results shown in Figure 2A indicate that *hpr1* and *tho2* did not affect the expression of *SIR4*, *CDC13*, or *RAP1*. The Rif2p level was only slightly decreased in these two mutants. Since a yeast strain with 9 repeats of Myc attached to the 3′ end of the chromosomal *RIF1* gene was used in our analysis, the effect of Myc-tagging on Rif1p was also analyzed. The *RIF1*-myc9 tagged allele caused similar, if not identical, effects on telomere length and telomere silencing to those observed in cells with the wild-type allele, implying that the tagged Rif1p is expressed and functional (Fig. 2B).

**Table 1:** The sizes and G+C contents of genes encoding telomere-associated proteins.

Gene name	ORF size	G+C content
*RIF1*	5751	36.24
*SIR4*	4077	37.23
*SIR3*	2937	36.50
*CDC13*	2775	43.93
*EST2*	2655	33.79
*RAP1*	2484	41.51
*PIF1*	2580	39.34
*EST1*	2100	36.00
*YKU80*	1890	39.52
*STN1*	1485	36.97
*YKU70*	1809	33.78
*SIR2*	1689	39.67
*TLC1*	1301	35.59
*RIF2*	1188	34.68
*EST3*	547	38.90
*TEN1*	483	35.60
*TEL1*	8364	35.80
*LacZ*	3069	56.21

It has been reported that the expression levels of yeast genes increase with the G+C contents in wild-type and both *hpr1* and *tho2* cells [44], [45]. The G+C contents of the genes encoding telomere-associated proteins were also surveyed (Table 1). Among a total of 16 genes analyzed, all of them showed a G+C content within the range of 34–44%, similar to the ∼40% average G+C content of all yeast genes. As a comparison, the *E. coli lacZ* gene has a G+C content of ∼56%. According to our findings, the expression of telomere-associated genes in *hpr1* and *tho2* cells did not appear to be affected by their G+C content (Fig. 2A).

To test if the reduction of Rif1p protein is specific to *hpr1* and *tho2* mutations, Rif1p protein expression was also determined in *mft1* and *thp2* cells. We discovered that the Rif1p protein level remained the same in *mft1* or *thp2* cells (Fig. 2C), indicating that the reduction in Rip1p levels is specific to *hpr1* and *tho2* mutations. This result is also consistent with our observation that only *hpr1* and *tho2* but not *mft1* or *thp2* cells showed long telomeres (Fig. 1A).

To determine whether the reduced Rif1p level was due to transcription defects caused by *hpr1* and *tho2* mutations, the RNA level of *RIF1* was analyzed. Total RNAs were prepared from yeast cells and hybridized with specific probes against *GCN1* (8,019 bps), *RIF1* (5,751 bps), *RIF2* (1,188 bps), and *TUB1* (1,460 bps) genes. As shown in Figure 2D, both *hpr1* and *tho2* mutations showed defects in transcribing the two long genes *GCN1* and *RIF1*. The effect is specific, as the transcript levels of short genes *RIF2* and *TUB1* were not affected by the mutations. Thus, our results are in agreement with the previous findings which suggest that *hpr1* and *tho2* mutations affect the transcription of long genes. Consequently, both the protein and RNA levels of *RIF1* were significantly reduced in these cells.

### *THO2* and *HPR1* share the same pathway of *RIF1* to regulate telomere length

**Figure 3:**
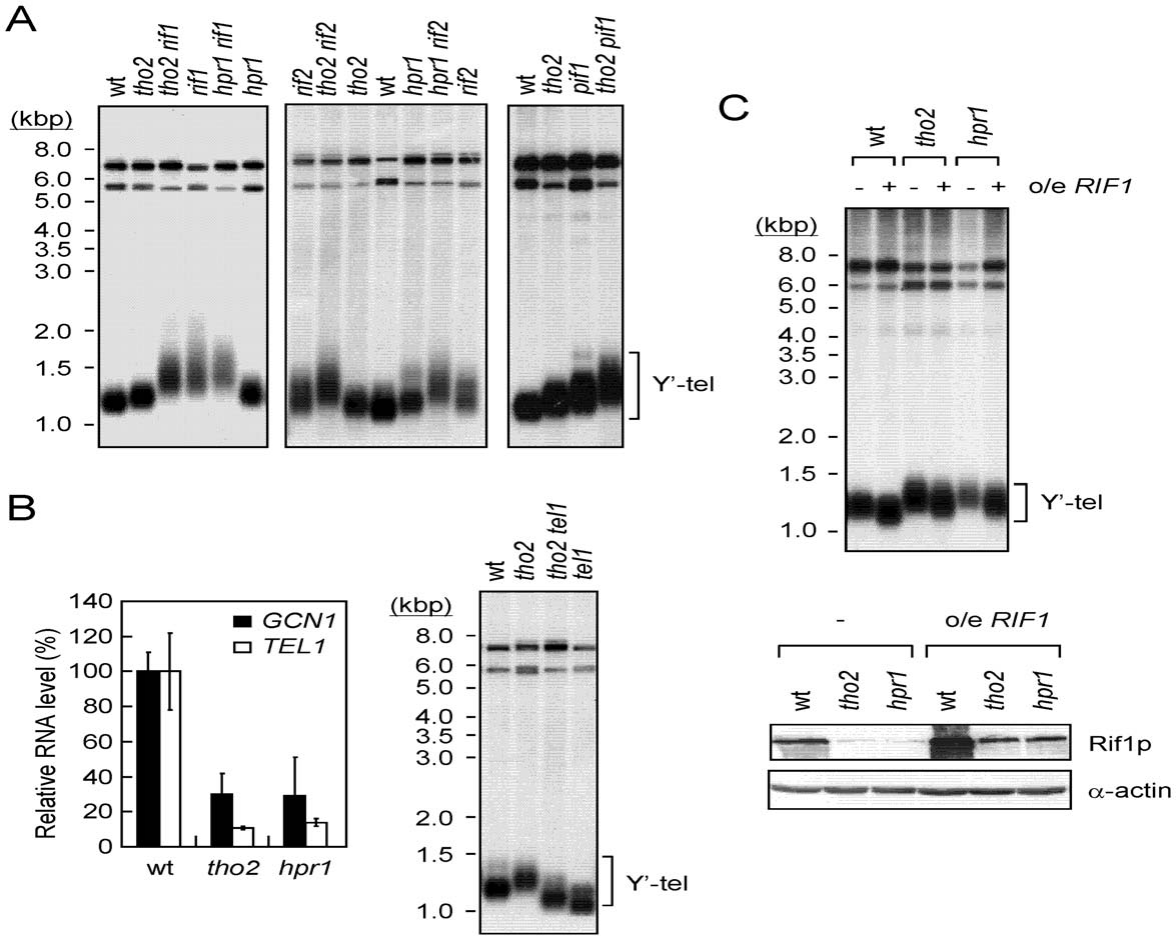
*THO2* and *HPR1* function in the same pathway as *RIF1* to regulate telomere length. (**A**) Yeast DNA from the indicated mutations was isolated and analyzed by Southern blotting assays using the Y′ element probe. (**B**) The decreased Tel1p level does not contribute to telomere lengthening in *tho2* and *hpr1* cells. Total mRNA from the indicated strains was isolated and analyzed for *GCN1* and *TEL1* RNA levels using real time RT-PCR (left panel). Results were presented as relative levels normalized to the wild-type expression level. The bars were standard deviations determined from three independent experiments. Yeast DNA from indicated strains was analyzed for telomere length (right panel). (**C**) Overexpressing Rif1p suppresses the telomere lengthening in *tho2* and *hpr1* cells. Wild-type, *tho2*, or *hpr1* yeast cells carrying plasmids pRS426 or pRS426-*RIF1* (o/e *RIF1*) were cultured at 30°C. Telomere lengths of these cells were then analyzed using Southern blotting assays (top panel). Immunoblotting analysis of the Rif1p level was also performed (bottom panel).

Given the role of *RIF1* as a negative regulator of telomere length and the finding that the deletion of *RIF1* in yeast causes elongation of telomere length [27], it is likely that the long telomeres observed in *hpr1* and *tho2* cells were due to the reduction of *RIF1* expression. This simple scenario predicts that the telomere lengths of *rif1 hpr1* or *rif1 tho2* cells should not be longer than that in the *rif1* cells. Indeed, the telomere lengths of cells carrying *rif1 hpr1* and *rif1 tho2* mutations were similar to those in the *rif1* cells (Fig. 3A). In comparison, we found that telomere lengths were further elongated when *rif2* was introduced into *hpr1* or *tho2* cells (Fig. 3A). Similarly, combinatorial effects were also observed when *pif1-m2* mutation was introduced into *tho2* cells (Fig. 3A). These results show that Hpr1p and Tho2p might affect telomere length through the same pathway as Rif1p.

It is also interesting to note that *TEL1* is a long gene (8,364 bps) and its expression level is decreased in *hpr1* cells [44]. Using real time RT-PCR, we found that the amount of *TEL1* RNA in *hpr1* or *tho2* cells was decreased to ∼10–15% of the wild-type level (Fig. 3B, left panel). In comparison, the RNA level of *GCN1* was also decreased in *hpr1* or *tho2* cells, consistent with our previous observation (Fig. 2 and 3B). Although Tel1p is not directly associated with telomeres, it participates in telomerase recruiting and its mutation has been shown to shorten telomeres [19]. To evaluate the contribution of *tel1* on telomere length maintenance in THO mutants, telomere length of *tel1 tho2* cells was determined. As shown in Figure 3B (right panel), telomere length in both wild-type and *tho2* mutant cells was decreased by the same extent after the introduction of a *tel1* mutation. The results suggest that although *TEL1* RNA level is also decreased in THO mutants, the observed telomere lengthening in *hpr1* or *tho2* cells is not due to the decreased Tel1p level.

Our model also predicts that increasing the Rif1p level should suppress the long telomere phenotype associated with *hpr1* and *tho2* mutations. Initial attempts to overexpress *RIF1* using a *cen* plasmid did not yield a detectable elevation of Rif1p levels in *hpr1* or *tho2* cells. Accordingly, there was no change in telomere length in these cells (data not shown). We then increased Rif1p levels by introducing a high-copy number 2 μ plasmid carrying *RIF1* into yeast cells. As shown in Figure 3C, increasing Rif1p reduced telomere length in wild-type cells. Long telomeres observed in *hpr1* and *tho2* cells were suppressed when Rif1p levels were brought back to the wild-type level. Significantly, there appeared to be a negative correlation between telomere lengths and the expression levels of Rif1p in the cells we examined. The results provide strong evidence supporting that the long telomeres observed in *hpr1* and *tho2* cells are caused by the reduction in the expression level of Rif1p.

### Enhancement of telomere silencing in *hpr1* and *tho2* cells

**Figure 4:**
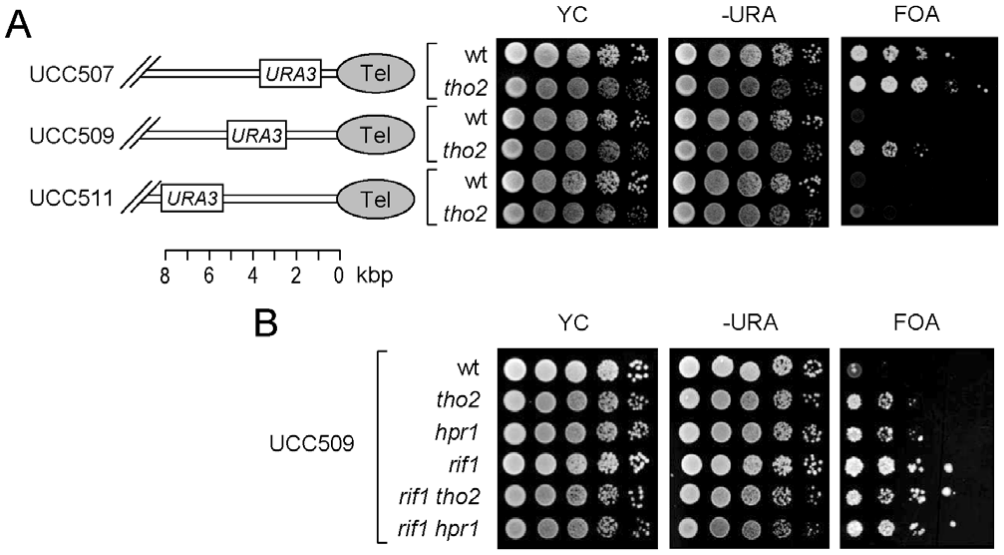
Deletion of THO complex components Hpr1p or Tho2p enhances the telomere position effect. (**A**) *tho2* enhances the telomere position effect. Wild-type or *tho2* cells with *URA3* at 1.1 kbp (UCC507), 2.5 kbp (UCC509), or 5.5 kbp (UCC511) from the telomere of chromosome VII–L were analyzed. Cells in 10-fold serial dilutions were spotted on YC, YC lacking uracil, or plates containing 5-FOA, and incubated until colonies formed. (**B**) *THO2* and *HPR1* function in the same pathway as *RIF1* to regulate the telomere position effect. Samples of serial dilutions of UCC509 cells with the indicated mutations were spotted on plates as indicated.

The function of *RIF1* on telomeres is not limited to telomere length regulation. It has been reported that *rif1* also enhances the telomere silencing effect [26], [46]. To test if mutations of THO components affect telomere silencing, expression of *URA3* at varying distances from telomeres was analyzed in strains carrying THO mutations. As shown in Figure 4A, stronger repression of *URA3* was observed in cells harboring a *tho2* mutation. The enhancement of *URA3* repression was observed in all three strains tested and was most obvious in UCC509. The effect of *hpr1* and *rif1* on telomere silencing was then evaluated using UCC509. We anticipated that *hpr1* would affect telomere silencing in a way similar to that of *tho2* mutation and that the addition of the *hpr1* or *tho2* mutation to the *rif1* mutant should not further enhance telomere silencing. The results shown in Figure 4B, *tho2* and *hpr1* showed similar enhancement of telomere silencing, ant the enhancement in both THO mutants was less than that conferred by the *rif1* mutation. The addition of the *tho2* or *hpr1* mutation had no further effect on telomere silencing in the *rif1* mutant. Thus, mutations on the two THO components also affect telomere silencing through a pathway mediated by *RIF1*.

### Overexpressing *SUB2* cannot restore the Rif1p level or telomere length in *tho2* and *hpr1* cells

**Figure 5:**
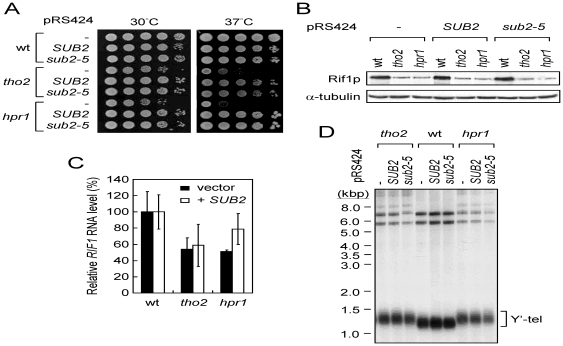
Overexpressing *SUB2* cannot restore the Rif1p level and telomere length in *tho2* and *hpr1* cells. (**A**) Overexpressing *SUB2* suppressed the growth defect of *tho2* or *hpr1* cells. Strains of the indicated genotypes derived from *THO2*/*tho2*, or *HPR1*/*hpr1* diploids carrying *SUB2* or *sub2-5* overexpressing plasmids were grown on YC plates at 30° or 37°C. (**B**) *SUB2 or sub2-5* overexpression did not restore the Rif1p level in *tho2* or *hpr1* cells. Immunoblotting assays were carried out as described. (**C**) *SUB2* overexpression did not restore the *RIF1* level in *tho2* or *hpr1* cells. Total mRNA from the indicated strains was isolated and analyzed for the *RIF1* RNA level using real time RT-PCR. Results were presented as relative levels normalized to the wild-type expression level. The bars were standard deviations calculated using data from three independent experiments. (**D**) *SUB2 or sub2-5* overexpression did not restore the telomere length in *tho2* or *hpr1* cells. Telomere length analyses were performed as previously described.

A high-copy suppressor screen using *lacZ* fused to a strong promoter has found that *SUB2* suppresses the transcriptional defect of *hpr1* [47]–[49]. *SUB2* encodes a DEAD-box RNA helicase that is involved in splicesosome assembly and mRNA exporting [50]–[52]. Sub2p binds to Yra1p and the THO complex to form a TREX (TRanscription/EXport) complex that couples elongation and exporting of mRNA [35], [52], [53]. To test whether overexpressing *SUB2* also recovers *RIF1* expression and restores telomere length in THO mutants, *tho2* and *hpr1* cells with a plasmid carrying *SUB2* or *sub2-5* were analyzed for Rif1p levels and telomere length. The *sub2-5* allele expresses a mutant Sub2p with a Q308R mutation near the helicase motif IV, which does not cause any detectable defects in splicing activity or spliceosome assembly [54]. However, the expression level of *sub2-5* is higher than that of wild-type *SUB2* in cells for unknown reasons [55]. As shown, overexpressing *SUB2* or *sub2-5* could rescue the growth defect of *tho2* and *hpr1* at 37°C (Fig. 5A), but did not restore the Rif1p level (Fig. 5B). Our results of real time RT-PCR experiments also showed that the *RIF1* RNA in both *tho2* and *hpr1* cells was not restored to the wild-type level by overexpressing *SUB2* (Fig. 5C). Consequently, telomere length in the *tho2* and *hpr1* cells remained long (Fig. 5D). Thus, while it is unclear why the Rif1p level cannot be restored through overexpressing *SUB2* or *sub2-5*, the results support our conclusion that the lowered Rif1p level is the cause for long telomeres in *tho2* and *hpr1* cells.

## Discussion

Telomere length homeostasis is mainly achieved through regulation of telomerase and telomere-associated proteins. Here we discovered that THO complex components Hpr1p and Tho2p also affect telomere length and telomere silencing. Long telomeres and enhanced telomere silencing were observed in *hpr1* and *tho2* cells. We also discovered that *RIF1* expression was greatly reduced in *hpr1* and *tho2* cells, which is in agreement with a previous observation that showed selective reduction of long transcripts by THO mutations [36]. Moreover, our genetic analyses using double mutations and overexpression demonstrated that telomere phenotypes observed in *hpr1* and *tho2* cells were caused by reduction of Rif1p levels. Thus, we have provided evidence that THO mutations exert their effects on telomeres through affecting the expression of Rif1p, the largest telomere-associated protein.

It has been shown that the *hpr1* and *tho2* mutations have the highest impact on gene expression and recombination among THO mutations [56]. Similarly, here we found that mutations on *HPR1* and *THO2* affected both telomere length and telomere silencing, whereas mutations on the other two THO components, Mft1p and Thp2p, did not affect Rif1p level and telomere lengths. Thus, it appears that the roles of Hpr1p and Tho2p are functionally distinct from those of Mft1p and Thp2p. Although the function of each component within the THO complex and the detailed mechanism underlying the differential impacts of individual THO mutations are not clearly defined, there appears to be a hierarchical difference within these components in terms of their *in vivo* biological relevance. Among these four components, the Tho2p and Hpr1p subunits are more important for telomere homeostasis.

Mutations of components of the THO complex cause a reduction in the transcription of yeast genes [34], [36]–[38], [45], [57]. Using the *E. coli lacZ* gene as a reporter, it has been shown that the level of gene transcription decreases with increasing gene length [39]. Further analysis has revealed that high G+C content might contribute to the reduced expression of *lacZ* [39]. Here we demonstrate a preferential reduction of *RIF1* expression in *hpr1* or *tho2* cells. The result is consistent with the prediction of preferential reduction of long genes in THO mutants. We did not find noticeable reduction in the expression of high G+C content genes in *hpr1* or *tho2* cells. It is possible that because the overall G+C contents of the genes encoding telomere-associated proteins are low, the effect of G+C content becomes less apparent. In any case, the G+C content did not appear to have a role in modulating the expression of genes encoding telomere-associated proteins in cells depleted of the THO complex.

A genome-wide expression analysis using microarray has showed that the *TEL1* level was significantly decreased in *hpr1* cells [44]. Our analysis also shows that the *TEL1* RNA levels are decreased in both *tho2* and *hpr1* cells. Because cells harboring a *tel1* mutation have short telomeres [19], a combinatory effect of telomere shortening and lengthening was observed in the *tho2 tel1* double mutant cells (Fig. 3B). This suggests that the telomere lengthening observed in *tho2* or *hpr1* cells is not due to the reduced *TEL1* RNA levels. It is possible that although *TEL1* expression is reduced in THO mutants, the amount of Tel1p is still sufficient to maintain its function on telomeres. In contrast, a ∼40–50% reduction of *RIF1* RNA level causes a ∼75–80% reduction of Rif1p level, which is sufficient to contribute to an increase of telomere length. The results implicate that the Rif1p level has to be tightly regulated to stably maintain the telomere length. Of note, although we found significant reduction of *RIF1* expression in both *tho2* and *hpr1* cells, *RIF1* was not identified among the repressed genes in THO mutants in the genome-wide expression analyses. The microarray analyses considered the genes with expression levels at least 1.5-fold above or below the wild-type values as significantly different [44]. It is possible that the ∼40–50% reduction of the *RIF1* RNA level in *tho2* and *hpr1* cells evaded the detection by microarray analyses.

Through a high-copy suppressor screen, it has been discovered that overexpressing *SUB2* suppressed both the growth and transcription defects of *tho2* and *hpr1* mutants [47]–[49]. It is surprising to us that the Rif1p levels were not restored by overexpressing *SUB2* or *sub2-5* in *tho2* and *hpr1* cells, although the growth defects were abrogated. The cause of the temperature-sensitive growth in *tho2* and *hpr1* cells is unclear. Given roles of the THO complex in transcription and mRNA export, it is possible that the growth defect is due to the impairment of bulk transcription-, mRNA export, or the generation of transcriptional byproducts upon the collapse of these gene expression processes. Elucidation of the involving molecular mechanism awaits the identification of target yeast genes affected by the THO complex mutations. Alternatively, it is also possible that due to a separation of function conferred by the *sub2* alleles, *SUB2* overexpression leads to suppression of temperature sensitivity but not telomere phenotypes. Indeed, it has been reported that *SUB2* overexpression in *tho2* cells only recovers ∼20–30% of the *lacZ* activity driven by a *GAL1* promoter [49], further supporting that *SUB2* might function differently in suppressing growth defect and telomeres. No matter what mechanism is used by Tho2p and Hpr1p in controlling gene expression, our results still clearly indicate that the lowered Rif1p level is the cause of long telomeres in *tho2* and *hpr1* cells.

## Materials and Methods

### Strains and plasmids

All of the strains in this study were constructed in the YPH499 (*MATa ura3-52 lys3-5 ade2-10 trp1-Δ63 his3-Δ200 leu2-Δ1*) background [58]. YPH499-derived strains carrying *SIR4*-TAP-*TRP1*, *tlc1Δ*::*LEU2*, *tho2Δ*::*HIS3*, *hpr1Δ*::*HIS3*, *mft1Δ*::*HIS3*, *thp2Δ*::*HIS3*, *tel1Δ*::*HIS3*, *rif1Δ*::*TRP1*, *rif2Δ*::*TRP1* and *pif1–m2* were constructed in this study or obtained from S.-C. Teng (National Taiwan University College of Medicine, Taipei, Taiwan). MS179 (Rif1-G8-myc9) and MS206 (Rif2-G8-myc9) yeast strains were kindly provided by V. A. Zakian (Princeton University). In these two strains, the chromosomal copies of *RIF1* and *RIF2* were tagged with 9-myc, respectively. A spacer with 8-glycine was also introduced in between *RIF1* or *RIF2* and the tag [59]. Both strains were back crossed four times to YPH499 to minimize differences in genetic backgrounds. UCC507, UCC509, and UCC511 strains were kindly provided by D. E. Gottschling [60]. In these strains, *URA3* was inserted at 1.2, 2.5, 5.5 kbp away from the left telomere of chromosome VII, respectively. Plasmid pRS426-RIF1-myc9 was generated by PCR amplification of the 3′ DNA fragment of RIF1-G8-myc9 using the genomic DNA of MS179 as the template and subsequent subcloning of the fragment into plasmid pRS426-RIF1 (2µ, *URA3*, D. Shore, University of Geneva, Sciences III, Geneva, Switzerland [26]). Plasmids pRS424-*SUB2* and pRS424-*sub2-5* were provided by E. Lahue [55].

### Telomere length determination

To determine telomere lengths, yeast DNA was prepared, digested with *Xho*I, and separated on 1% agarose gels. Next, DNA fragments were transferred to a Hybond N^+^ paper (Amersham) for hybridization using a random primed Y′ DNA probe [61].

### Immunoblotting analysis

Immunoblot analysis was carried out according to standard procedures using ECL detection (Perkin Elmer). Polyclonal antibodies against Rap1 were kindly provided by S.-C. Teng. Polyclonal antibodies against Cdc13(1–252)p were raised in rabbits [62]. Anti-α-tubulin (T9026) and anti-actin (MAB1501) antibodies were purchased from Sigma-Aldrich and Millipore, respectively. The anti-myc antibody (for Rif1-G8-myc9 and Rif2-G8-myc9) was purchased from LTK-Biolab. The anti-TAP antibody (CAB1001, for Sir4-TAP) was purchased from Open Biosystems. Horseradish peroxidase-conjugated donkey anti-rabbit or sheep anti-mouse antibodies (Amersham) were used as the secondary antibodies.

### Northern Blotting analysis

Yeast cells were grown to an OD_600_ of 0.6 and then RNA was extracted using the hot phenol extraction protocol. Briefly, yeast cells were harvested by centrifugation and quickly resuspended in cold TES (10 mM Tris pH 7.5, 10 mM EDTA, 0.5% SDS). Equal volumes of acid phenol (Phenol ∶ Chloroform = 5 ∶ 1, pH 4.5, Sigma) were added and the samples were incubated at 65°C for 60 min. Acid phenol extraction was repeated several times until the interface was clear. The extracted RNA was extracted with equal volumes of CHCl_3_ and precipitated by ethanol. Ten µg of the extracted RNAs were separated by 1.2% formaldehyde agarose gels, transferred onto Hybond N^+^ membranes (Amersham), and hybridized with DNA probes prepared by random priming. The primers used to prepare PCR amplified DNA probes were: Rif1 forward (5′-ATCGAATTGATCAGCATATCCTCCGTAGGTCTCAACACGA-3′), Rif1 reverse (5′-GCTACACTTTTTGTAACCGG-3′), Rif2 forward (5′-ATGGAGCATGTAGATTCCGATTTTGCACCTATAAGGAGAT-3′), Rif2 reverse (5′-GAAACTTACTTAAGCTCGGA-3′), Tub1 forward (5′-AGTATTAATGGTATGTTCGATTTGCCCGTCCAGGCTAGAT-3′), Tub1 reverse (5′-GTAAATAGCCCTTGGAACGA-3′), Gcn1 forward (5′-GCGAATCACACGTGTCTAAAAGAGTTCCCTTTTTGCAAGA-3′), and Gcn1 reverse (5′-GCGACACAAAAATCAAGAAA GA-3′).

### Real time RT-PCR analysis

Five micrograms of RNA were first treated with RNase-free DNase (Qiagen #79254) at 37°C for 30 min. Reverse transcription of the RNA was conducted using the RevertAid M-MuLV reverse transcriptase (Fermentas #EP0442) with oligo-dT as the primer. Real time PCR analyses were conducted using the ABI Step one real-time PCR system. Primer pairs used in this analysis were: GCN1 (5′-TGCGTGATCCTGTTATCCCA-3′, 5′-CGACAGCCCATATTTCTCTTTCATCTTCAAATCTGTCACT-3′), RIF1 (5′-ATTCACCTCCTCGAATGACA-3′, 5′-AGTTTCTTCTTTCTTCCACAGATATTCCGTTCAAGTCGCC-3′), TEL1 (5′-AGGCGGAGTTGTGAAAGAGTTTACGCAGTA-3′, 5′-AAATTTGATGGATCCGTGGCTTGCTGAATC-3′), and TUB1 (5′-AAGAAGACCGTCCAATTGGT-3′, 5′-GGCACCCACTTCGATGTAATCTCTTTCTAAAGCAGCCAAA-3′). The *TUB1* RNA level was used as an internal control in each set of the experiments.

### Assay for telomere position effect

Strains UCC507, UCC509, and UCC511 were used to evaluate the effect of telomere silencing. These strains were crossed with YPH500 carrying *tho2*, *hpr1*, or *rif1* mutations. The resulting diploid strains were induced into meiosis and the resulting tetrads were dissected. Haploid strains with *URA3* placed near the telomere and *tho2*, *hpr1*, or *rif1* mutations were then selected. These cells were streaked on YC plates and grown for 3 days at 30°C. Colonies were resuspended in water, and aliquots of different dilutions were spotted on YC plates, YC plates lacking uracil or plates containing 5-FOA. Plates were incubated at 30°C until colonies formed. Usually, it takes 3 days for colonies to form on YC plates, and it takes 6–7 days on plates containing 5-FOA.
