# Correction: Depleting Components of the THO Complex Causes Increased Telomere Length by Reducing the Expression of the Telomere-Associated Protein Rif1p

**DOI:** 10.1371/annotation/2e35aeeb-4f49-4957-931f-9d942a856e42

**Published:** 2012-05-04

**Authors:** Tai-Yuan Yu, Chen-Yi Wang, Jing-Jer Lin

There was an error in the affiliations. The correct affiliations are:

1 Institute of Biopharmaceutical Sciences, National Yang-Ming University, Taipei, Taiwan, R.O.C.,

2 Institute of Biochemistry and Molecular Biology, National Taiwan University College of Medicine, Taipei, Taiwan, R.O.C. 

